# Classification of Transcription Boundary-Associated RNAs (TBARs) in Animals and Plants

**DOI:** 10.3389/fgene.2018.00168

**Published:** 2018-05-14

**Authors:** Dongliang Yu, Xiaoxia Ma, Ziwei Zuo, Huizhong Wang, Yijun Meng

**Affiliations:** College of Life and Environmental Sciences, Hangzhou Normal University, Hangzhou, China

**Keywords:** transcription boundary-associated RNAs (TBARs), promoter-associated RNAs (PARs), terminus-associated RNAs (TARs), divergent transcription, transcription start site (TSS), transcription termination site (TTS), polymerase (Pol) pausing, gene looping

## Abstract

There is increasing evidence suggesting the contribution of non-coding RNAs (ncRNAs) to the phenotypic and physiological complexity of organisms. A novel ncRNA species has been identified near the transcription boundaries of protein-coding genes in eukaryotes, bacteria, and archaea. This review provides a detailed description of these transcription boundary-associated RNAs (TBARs), including their classification. Based on their genomic distribution, TBARs are divided into two major groups: promoter-associated RNAs (PARs) and terminus-associated RNAs (TARs). Depending on the sequence length, each group is further classified into long RNA species (>200 nt) and small RNA species (<200 nt). According to these rules of TBAR classification, divergent ncRNAs with confusing nomenclatures, such as promoter upstream transcripts (PROMPTs), upstream antisense RNAs (uaRNAs), stable unannotated transcripts (SUTs), cryptic unstable transcripts (CUTs), upstream non-coding transcripts (UNTs), transcription start site-associated RNAs (TSSaRNAs), transcription initiation RNAs (tiRNAs), and transcription termination site-associated RNAs (TTSaRNAs), were assigned to specific classes. Although the biogenesis pathways of PARs and TARs have not yet been clearly elucidated, previous studies indicate that some of the PARs have originated either through divergent transcription or via RNA polymerase pausing. Intriguing findings regarding the functional implications of the TBARs such as the long-range “gene looping” model, which explains their role in the transcriptional regulation of protein-coding genes, are also discussed. Altogether, this review provides a comprehensive overview of the current research status of TBARs, which will promote further investigations in this research area.

## Introduction

In the initial phase of genome sequencing, annotated protein-coding genes were thought to be a major contributor to the development of phenotypic and physiological complexity of organisms. However, this viewpoint was challenged soon afterwards. The application of high-throughput sequencing (HTS) in transcriptomics studies uncovered the intriguing phenomenon in which transcription signals were detected across the entire genomes (Encode Project Consortium et al., 2007). Notably, most of these signals originated from non-coding loci but not from protein-coding genes. Subsequent studies have revealed a positive correlation between the diversity of non-coding RNAs (ncRNAs) and the complexity of organisms (Amaral and Mattick, 2008; Amaral et al., 2008). Considering the emerging biological importance of ncRNAs, continuous research efforts have led to the identification and characterization of ncRNAs in multiple organisms. Although a large proportion of ncRNAs resides within intergenic regions, some reside within protein-coding genes, such as antisense ncRNAs (He et al., 2008) and intronic ncRNAs (Brown et al., 2008; Meng et al., 2011; Meng and Shao, 2012). Additionally, some of the ncRNAs have been identified near the promoter regions or the transcription termini of protein-coding genes (Gingeras, 2007; Kapranov et al., 2007).

In this review, we provide a brief summary of the discovery of promoter-associated RNAs (PARs) in budding yeast (*Saccharomyces cerevisiae*) (Davis and Ares, 2006; Neil et al., 2009; Xu et al., 2009), fruit fly (*Drosophila melanogaster*), chicken (*Gallus gallus*) (Taft et al., 2009a), mouse (*Mus musculus*) (Kapranov et al., 2007; Seila et al., 2008; Affymetrix Encode Transcriptome Project and Cold Spring Harbor Laboratory Encode Transcriptome Project, 2009; Flynn et al., 2011), human (*Homo sapiens*) (Han et al., 2007; Kapranov et al., 2007; Preker et al., 2008; Affymetrix Encode Transcriptome Project and Cold Spring Harbor Laboratory Encode Transcriptome Project, 2009; Pickrell et al., 2010), *Arabidopsis thaliana* (Chekanova et al., 2007; Wang et al., 2011; Ma et al., 2017), bacteria (Yus et al., 2012), and archaea (Zaramela et al., 2014). Additionally, the discovery of terminus-associated RNAs (TARs) in human (Kapranov et al., 2007, 2010; Taft et al., 2009a; Yue et al., 2010; Valen et al., 2011; Schwalb et al., 2016), mouse (Kapranov et al., 2007), fission yeast (*Schizosaccharomyces pombe*), and *Arabidopsis* (Ma et al., 2017) has also been introduced. Since promoters and terminators define the transcription boundaries of protein-coding genes, PARs and TARs are referred to as transcription boundary-associated RNAs (TBARs). Additionally, we summarize the biogenesis pathways of some of the TBARs and a few mechanistic models of TBAR biogenesis including divergent transcription and RNA polymerase (Pol) pausing. We also summarize recent progress in the functional studies conducted on TBARs, followed by in-depth discussions. Interestingly, PARs have been functionally implicated in transcription termination, whereas TARs are potentially involved in transcription initiation. The interactive relationship between transcription initiation and termination has been depicted using long-range regulatory models, such as “gene looping” (Lykke-Andersen et al., 2011; Andersen et al., 2013). Finally, we propose a preliminary nomenclature system for TBARs according to specific classification criteria and emphasize the importance of a uniform annotation system for TBAR research.

## PARs in Animals: Discovery, Biogenesis, and Functions

In eukaryotes, the transcription of protein-coding genes includes two primary steps: initiation and elongation. Each step is under strict surveillance. However, each time transcription is initiated from the transcription start site (TSS) of a protein-coding gene, the final output is not always a full-length coding sequence. This is because various kinds of genetic and epigenetic factors, such as binding sites of RNA Pol II or transcription factors (TFs) and chromatin modifications, interrupt transcription initiation and/or elongation (Li et al., 2007; Razin et al., 2011). Premature termination of transcription is a widespread phenomenon that results in aberrant transcripts. As a result of frequent transcription pausing, diverse ncRNAs have been discovered near the promoter regions. Although such ncRNAs have been referred to by different names, here they are referred to as PARs.

In a pioneering study, Core et al. (2008) employed global run-on sequencing (GRO-Seq) to investigate the density of transcriptionally engaged RNA polymerase enzymes on a genome-wide scale. This study identified a novel species of PARs in human lung fibroblasts. However, because this study used an older generation sequencing platform with short read-length (maximum 33 nt), data on the length of PARs was not provided. These PARs are enriched within -250 bp to +50 bp of the TSSs of certain protein-coding genes. Because GRO-Seq is capable of evaluating promoter-proximal pausing on all genes, it has been suggested that these PARs are derived from RNA Pol II-dependent divergent transcription and promoter-proximal pausing (Core et al., 2008). Subsequent studies have uncovered PARs in different species. Because of recent advances in sequencing platforms, more information is available on the sequence features of PARs, especially their length distribution. In this review, we propose that, according to their sequence length, PARs should to be divided into two classes: promoter-associated long RNAs (PALRs; >200 nt) and promoter-associated small RNAs (PASRs; <200 nt).

Promoter-associated small RNAs have been reported in human and mouse (Kapranov et al., 2007; Affymetrix Encode Transcriptome Project and Cold Spring Harbor Laboratory Encode Transcriptome Project, 2009). TSS-associated RNAs (TSSaRNAs) reported in mouse (Seila et al., 2008) and transcription initiation RNAs (tiRNAs) in human, mouse, chicken and *Drosophila* (Taft et al., 2009a) have also been categorized as PASRs. Based on these studies, it can be concluded that weak expression is a common feature of PASRs. A number of PARs are exosome-specific substrates with short half-lives (Wyers et al., 2005; Davis and Ares, 2006; Chekanova et al., 2007; Preker et al., 2008). The divergent distribution of many PARs near the TSSs is correlated with bidirectional transcription activity of RNA Pol II. Functional studies have connected some of the PARs with transcriptional activation or repression of their target genes (Li et al., 2006; Janowski et al., 2007; Morris et al., 2008; Turunen et al., 2009; Huang et al., 2010; Kassab et al., 2015; Li et al., 2016; Uesaka et al., 2017), indicating that transcription can be modulated not only during elongation but also at initiation (Saunders et al., 2006; Muse et al., 2007; Zeitlinger et al., 2007; Core et al., 2008). In the following sections, we describe the previously reported PARs in animals and their biogenesis pathways and biological functions.

### PALRs in Animals

#### Different Classes of PALRs

As summarized in **Table 1**, different classes of PALRs have been reported in several organisms, including promoter-associated transcripts in human (Han et al., 2007) and yeast (Davis and Ares, 2006), promoter upstream transcripts (PROMPTs) in human (Preker et al., 2008, 2011), upstream antisense RNAs (uaRNAs) in mouse (Flynn et al., 2011), stable unannotated transcripts (SUTs) and cryptic unstable transcripts (CUTs) in yeast (Wyers et al., 2005; Neil et al., 2009; Xu et al., 2009), and other unnamed PALRs in human and mouse (Kapranov et al., 2007; Affymetrix Encode Transcriptome Project and Cold Spring Harbor Laboratory Encode Transcriptome Project, 2009). Till date, only a few studies have been conducted on the biogenesis pathways and modes of action of PALRs. Thus, it is unclear whether these PALRs, with distinct nomenclatures, belong to the same or different ncRNA species. It is well known that divergent transcription of active promoters is the major mechanism involved in the biogenesis of PALRs (Sigova et al., 2013). Among the above-mentioned PALRs, PROMPTs are relatively well studied, especially for their sequence characteristics, biogenesis and biological roles. Therefore, we describe the PROMPTs in detail in the sections below.

**Table 1:** List of the currently identified TBARs (transcription boundary-associated RNAs).

TBAR species			Discovery	Length	Reference
PARs	PALRs	PaRNAs	*H. sapiens* and *S. cerevisiae*	200–500 nt	Davis and Ares, 2006; Han et al., 2007
		PROMPTs	*H. sapiens*	Hundreds of nt	Preker et al., 2008; Preker et al., 2011
		UaRNAs	*M. musculus*	40–1,100 nt	Flynn et al., 2011
		SUTs and CUTs	*S. cerevisiae*	200–500 nt	Wyers et al., 2005; Neil et al., 2009; Xu et al., 2009
		UNTs	*A. thaliana*	–	Chekanova et al., 2007
		PALRs	*H. sapiens* and *M. musculus*	–	Kapranov et al., 2007; Affymetrix Encode Transcriptome Project and Cold Spring Harbor Laboratory Encode Transcriptome Project, 2009
	PASRs	PASRs	*H. sapiens, M. musculus* and *A. thaliana*	19–200 nt	Kapranov et al., 2007; Affymetrix Encode Transcriptome Project and Cold Spring Harbor Laboratory Encode Transcriptome Project, 2009; Wang et al., 2011; Ma et al., 2017
		TSSaRNAs	Eukaryotes, bacteria, and archaea	16–146 nt	Seila et al., 2008; Yus et al., 2012; Park et al., 2014; Zaramela et al., 2014
		TiRNAs	*H. sapiens*, *M. musculus*, *G. gallus*, and *D. melanogaster*	13–28 nt	Taft et al., 2009a
TARs	TALRs	TALRs	*H. sapiens*	∼3,100 nt	Yue et al., 2010
	TASRs	TASRs	*H. sapiens*, *M. musculus*, *S. pombe*, and *A. thaliana*	19–200 nt	Kapranov et al., 2007, 2010; Taft et al., 2009a; Leng et al., 2014; Schwalb et al., 2016; Ma et al., 2017
		TTSaRNAs	*H. sapiens*	22–24 nt	Valen et al., 2011

*TBARs, transcription boundary-associated RNAs; PARs, promoter-associated RNAs; TARs, terminus-associated RNAs; PALRs, promoter-associated long RNAs; PASRs, promoter-associated small RNAs; TALRs, terminus-associated long RNAs; TASRs, terminus-associated small RNAs; paRNAs, promoter-associated RNAs; PROMPTs, promoter upstream transcripts; uaRNAs, upstream antisense RNAs; SUTs, stable unannotated transcripts; CUTs, cryptic unstable transcripts; UNTs, upstream non-coding transcripts; TSSaRNAs, transcription start site-associated RNAs; tiRNAs, transcription initiation RNAs; TTSaRNAs, transcription termination site-associated RNAs.*

#### Discovery of PROMPTs

Promoter-associated RNAs have short half-lives, as they undergo rapid exosome-mediated degradation, which interferes with their detection. To overcome this limitation, Preker et al. (2008, 2011) used human HeLa cells without exosome activity for the identification of short-lived PARs using oligodT primers. Results of tiling microarray analysis revealed 5′ capped and 3′ adenylated transcripts upstream of the TSSs of transcriptionally active genes (Preker et al., 2008, 2011). These transcripts were several hundred nucleotides in length and were named as PROMPTs.

Unlike PARs discovered by Core et al. (2008), PROMPTs originate from the region within -2,500 bp to -500 bp upstream of the TSS. Moreover, the transcription of PROMPTs can proceed in both directions (Preker et al., 2008). It is widely accepted that divergent transcription is an inherent feature of most of the active promoters (Beck and Warren, 1988; Neil et al., 2009; Xu et al., 2009). Moreover, the production of PROMPTs is highly dependent on activity of promoters of neighboring genes (Preker et al., 2008). Thus, the bidirectional transcription of PROMPTs reflects the universality of divergent transcription of highly active genes, which links bidirectional transcription to gene activity (Core et al., 2008; Nechaev and Adelman, 2011).

#### Functions of PROMPTs

Functional studies suggest that bidirectional transcription plays an important role in improving the accessibility of chromatin regions for the binding of TFs (Gilchrist et al., 2008; Seila et al., 2008). Notably, some of the divergently transcribed PARs are involved in Argonaute (AGO)-dependent (Han et al., 2007) or -independent (Wang et al., 2008) gene silencing pathways, indicating a potential regulatory role of PROMPTs in gene transcription. The SR proteins facilitate the function of promoter-proximal nascent RNA in transcription pause release (Ji et al., 2013). Additionally, some of the PROMPTs are enriched within the promoter regions with a high CpG content, and these PROMPTs affect the DNA methylation density of the promoters (Preker et al., 2008). Sequence motif analysis has shown that 3′ poly(A) signals are more abundant in regions upstream of the promoter than in those downstream of the promoter. These functional poly(A) signals are involved in the rapid degradation of PROMPTs transcribed in the antisense direction upstream of the associated genes. Thus, the rapid decay of upstream PROMPTs enables efficient elongation of downstream transcripts, which enforces the promoter orientation of protein-coding genes (Ntini et al., 2013). Moreover, if the synthesis of PROMPTs is stalled very early within the TSS-proximal regions, small TSSaRNAs are produced. Small TSSaRNAs are included in the category of PASRs and are discussed below.

### PASRs in Animals

#### Discovery of PASRs

Promoter-associated small RNAs (sRNAs) identified in human and mouse have been previously named as PASRs (Calabrese et al., 2007; Kapranov et al., 2007). These sRNAs are 20–200 nt in length and originate from the region within -400 bp to +400 bp of the TSS. TSSaRNAs are a type of PASRs that have been identified in a broad spectrum of organisms, including eukaryotes (Seila et al., 2008; Park et al., 2014), bacteria (Yus et al., 2012), and archaea (Zaramela et al., 2014). The length of TSSaRNAs differs between species. For example, in the archaea *Halobacterium salinarum*, the length of TSSaRNAs ranges from 16 to 146 nt, with a median size of 27 nt (Zaramela et al., 2014). In bacteria, the length of TSSaRNAs varies within a narrow range; in *Escherichia coli* and *Mycoplasma pneumonia*, the length of TSSaRNAs varies from 33 to 40 nt and 35 to 55 nt, respectively (Yus et al., 2012). In murine embryonic stem cells, TSSaRNAs vary in size from 20 to 90 nt. These TSSaRNAs are highly enriched in the region spanning from -250 bp to +50 bp of the TSS (Seila et al., 2008). Thus, sequence features of mouse TSSaRNAs are similar to those described above. Indeed, TSSaRNAs were occasionally confused with the PASRs (Affymetrix Encode Transcriptome Project and Cold Spring Harbor Laboratory Encode Transcriptome Project, 2009). Another class of PASRs comprises tiRNAs; these are highly conserved in higher metazoans, such as human, chicken, and fruit fly. Similar to the above two PASR classes, tiRNAs are enriched within the region spanning from -60 bp to +120 bp relative to the TSS. However, additional features specific to the tiRNAs have been reported, including their size distribution and GC content (Taft et al., 2009a). The size of tiRNAs varies from 13 to 28 nt, and a significant proportion of these are 18nt in length. Additionally, tiRNAs are GC-rich and show a strong strand bias toward the neighboring TSSs for their biogenesis.

#### Biogenesis of PASRs

The biogenesis of the three types of PASRs described above is Dicer-independent (Calabrese et al., 2007; Seila et al., 2008; Taft et al., 2009a), indicating that the non-canonical sRNA processing pathway(s) might be responsible for their production. Two models have been proposed for the biogenesis of PASRs (Lenhard et al., 2012). The first model is called “backtracking and excision” (Shaevitz et al., 2003; Seila et al., 2009; Taft et al., 2009a,b, 2010; Nechaev et al., 2010). In this model, the elongating RNA Pol II is stalled, inducing it to backtrack toward the upstream TSS after encountering the downstream nucleosome, resulting in a nascent transcript with a short 3′ exposed region. Subsequently, the exposed region is cleaved by the transcription elongation factor SII, resulting in PASR biogenesis. However, *in vitro* experiments have shown that the pausing and backtracking of Pol II is used to generate 6–14 nt fragments (Lenhard et al., 2012); however, this length is much shorter than that of PASRs. Therefore, further investigation is needed to identify a plausible model. The second model proposed for PASR biogenesis is the “Pol II pausing” model, which is also described as “unsuccessful Pol II elongation followed by RNA degradation” (Buckley et al., 2014; Jonkers et al., 2014). In this model, the RNA elongation complex is stalled at the initiation stage. Pol II pausing results in a nascent transcript without the 5′ cap, thus rendering it susceptible to rapid decay starting from its 5′ end. Only a short RNA sequence physically covered by Pol II is protected from degradation (Valen et al., 2011). The size of this short RNA sequence varies from17 to 22 nt, which fits well within the median size of PASRs. Notably, the above two models are probably mutually non-exclusive, since different PASR classes may use distinct pathways for their biogenesis.

#### Functions of PASRs

Recent studies have revealed valuable functional implications of PASRs. Depending on the association with certain long non-coding RNAs (lncRNAs) and protein factors, some of the PASRs target specific promoter regions of genes for epigenetic modifications that affect gene transcription (Hamazaki et al., 2017). However, altered transcriptional activity of target genes, in turn, causes fluctuations in PASR biogenesis. Thus, a feedback regulatory loop is established between PASRs and their target genes (Yan and Ma, 2012).

TSSaRNAs have been shown to play an important role in the tissue-specific epigenetic regulation of transcription initiation of target genes (Seila et al., 2008; Taft et al., 2009a, 2011; Henriques et al., 2013). In bacteria, TSSaRNAs are involved in the assembly of functional complexes that enable full-length transcription of their target genes, thus avoiding immature transcription initiation (Yus et al., 2012). The tiRNAs are produced from the promoter regions of highly expressed genes. These sRNAs are associated with chromatin marks representative of active transcription (Taft et al., 2009a, 2011). Specifically, approximately 96% of tiRNAs discovered in human embryonic stem cells overlap with histone H3K4 methylation marks (Taft et al., 2009a). Notably, some of these chromatin marks are generated via the tiRNA-dependent pathway and play important biological roles. For example, in both human and mouse, some of the tiRNAs induce local epigenetic modifications that modulate the localization of TFs that bind to CCCTC motifs (Taft et al., 2011).

## TARs in Animals: Discovery, Biogenesis, and Functions

In addition to the PARs, transcription signals have also been detected at the 3′ ends of protein-coding genes (Wei et al., 2011) (**Table 1**). In contrast to PARs, reports on TARs, especially terminus-associated long RNAs (TALRs), are limited. In one case of TALRs, Yue et al. (2010) discovered several sense ncRNAs of ∼3,100 nt at the 3′ end of a *progesterone receptor* gene. In another study using human K562 cells, Schwalb et al. (2016) detected transient RNAs downstream of the poly(A) sites using transient transcriptome sequencing (TT-Seq), although the length of these transient RNAs was not described in detail.

The terminus-associated small RNAs (TASRs) ranging from 22 to 200 nt in length are highly conserved in human and mouse (Kapranov et al., 2007, 2010). Traces of sRNAs at the 3′ ends of many animal genes have been reported (Taft et al., 2009a). In fission yeast, the sense TASR snR49 has been detected in the 3′ region of the ribosomal protein-coding gene *RPL26* (Leng et al., 2014). Another type of TASRs has been referred to as transcription termination site-associated RNAs (TTSaRNAs) (Valen et al., 2011). The enrichment of AGO-associated sRNAs within the 3′ untranslated regions (UTRs) of protein-coding genes has been observed in human (Valen et al., 2011); these TTSaRNAs typically vary in size from 22 to 24 nt.

Like PASRs, the genomic distribution of TASRs does not indicate a strand-specific bias. Some of the TASRs with non-genomically encoded 5′ poly(U) tails have been reported in human cells (Kapranov et al., 2010). Because these TASRs are antisense to poly(A)-tailed transcripts and are close to the transcription termination sites, these are suggested to be synthesized via an as-yet-unidentified RNA-dependent RNA polymerase (RDR)-dependent pathway. Another mechanism of TASR biogenesis has been reported for the 3′ sense terminus-associated ncRNA snR49 (Leng et al., 2014). Results of scanning deletion analysis within the promoter region reveal that the transcription of snR49 is dependent on the promoter activity of the upstream gene *RPL26*. Similarly, the transcription of another 3′ sense terminus-associated ncRNA, snR93, relies on the promoter activity of the upstream *RPL29* gene. These data suggest that this kind of transcriptional regulation is a conserved mechanism underlying TASR biogenesis (Leng et al., 2014).

To investigate the regulatory roles of TARs overlapping the 3′ termini of protein-coding genes in the human genome, Younger and Corey (2011) designed small duplex RNAs called “microRNA (miRNA) mimics,” which were perfectly complementary to the TARs. The targeting of TARs by “miRNA mimics” affects upstream gene transcription in a dose-dependent manner, highlighting a previously unappreciated role of gene termini and their associated TARs in transcriptional regulation (Younger and Corey, 2011). On the other hand, artificial constructs expressing 3′ UTRs containing miRNA-binding sites act as miRNA sponges (Thomson and Dinger, 2016), indicating that some of the TALRs might function as endogenous miRNA sponges. Another widely accepted model called “gene looping” elucidates TAR-mediated interaction between the 5′ promoters and 3′ terminators. This model provides an unconventional view of transcriptional regulation on a long-range scale (O’Sullivan et al., 2004; Tan-Wong et al., 2008; Tiwari et al., 2008; Yue et al., 2010). The long-range “gene looping” brings the two ends of a gene in close proximity. The sRNAs complementary to the 3′ sense TALRs recruit AGO2 and other protein factors to the 5′–3′ interaction region of the gene, thus modulating its transcription initiation (Yue et al., 2010). This “gene looping” also provides a long-range scaffold, thus enabling communication between endogenous TASRs and promoters of upstream genes (Younger and Corey, 2011).

## TBARs: The Emerging RNA Species in Plants

### Discovery of Plant TBARs

Transcription boundary-associated RNAs have also been discovered in plants, although most of the reports are related to PARs. In *Arabidopsis*, a striking association of UNTs with promoters of many protein-coding genes has been observed (Chekanova et al., 2007). In yeast, characteristics of UNTs are similar to those of CUTs (Wyers et al., 2005; Neil et al., 2009; Xu et al., 2009). For example, both UNTs and CUTs are weakly expressed, 3′ poly(A)-tailed, and degraded via the exosome-mediated pathway. Notably, the 5′ ends of UNTs are coincident with those of full-length mRNAs transcribed from the identical promoters.

A survey of PASRs in metazoa and *Arabidopsis* has shown that, unlike metazoans, PASR-like peak is not detectable within the promoter regions of *Arabidopsis* genes (Taft et al., 2009b). Data of this survey also suggest that 18-nt tiRNAs are absent in plants. There are two possible explanations for these observations. First, it is possible that the tiRNA biogenesis pathway either has been lost or never existed in *Arabidopsis*. Second, it is possible that once tiRNAs are produced, they are subjected to rapid degradation (Taft et al., 2009b). The presence of PARs and TARs in *Arabidopsis* has been revisited using many more HTS data sets (Wang et al., 2011). Unlike the previous study (Taft et al., 2009b), Wang et al. (2011) detected PASR peaks in regions surrounding the TSSs of non-transposable element (TE) genes. However, in these data sets, a total of 17,000 non-TE genes were treated as a whole group for PASR signal detection, and an in-depth investigation was not performed. Therefore, a detailed list of genes producing PASRs is not available. Recently, we identified hundreds of protein-coding genes in *Arabidopsis* with detectable PASR signals (Ma et al., 2017). Similar to the result of Taft et al. (2009a), the 18-nt tiRNA-like PASRs were also rarely detected in our study. Some of the PASRs accumulated in a tissue-specific manner. Additionally, we observed TASR peaks surrounding the transcription termini of many protein-coding genes (Ma et al., 2017). Both PASRs and TASRs vary in length from 23 to 24 nt and preferentially start with either an adenine (A) or a uracil (U) residue at their 5′ ends.

### Biogenesis of Plant TBARs

In *Arabidopsis*, PASRs are located either in upstream or downstream regions of the TSSs (Wang et al., 2011; Ma et al., 2017). In animals, divergent transcription (Beck and Warren, 1988; Core et al., 2008; He et al., 2008; Seila et al., 2008, 2009; Flynn et al., 2011) and bidirectional promoters (Morris et al., 2008; Xu et al., 2009; Wei et al., 2011; Uesaka et al., 2014) have been proposed as the mechanisms of PASR biogenesis. Whether these models explain the biogenesis of PASRs in plants needs further investigation. There is no clear model for the biogenesis of TASRs in animals. It has been suggested that some of the TASRs, especially those that areantisense, are generated through a specific RDR-dependent pathway. Our study in *Arabidopsis* shows that the accumulation of some TBARs, including PASRs and TASRs, is highly dependent on the activities of RNA Pol IV, RDR2/6, and DCL2/3/4 (Ma et al., 2017).

According to the distribution patterns of TSSs in vertebrates, gene promoters are classified into two categories: sharp and broad. A sharp promoter always has a single predominant TSS, whereas a broad promoter usually has an array of almost equivalent TSSs (Valen et al., 2011; Danino et al., 2015). Interestingly, sharp promoters generate fairly narrow PASR peaks, whereas broad promoters generate PASRs with a much wider distribution range. Notably, in *Arabidopsis*, both sharp and broad PASR peaks are observed on different gene promoters (Ma et al., 2017). Thus, investigating the relationship between the promoter type and PASR peak shape in plants will be useful.

### Functions of Plant TBARs

Few studies have been conducted on the regulatory roles of TBARs in plants. However, studies in animals provide valuable hints on the functions of TBARs in plants. Using the human *PR* gene as a model, Core et al. (2008) have shown that small duplex RNAs complementary to the ncRNAs originating from the promoter or terminus of the *PR* gene can efficiently regulate gene transcription. Theoretically, manually designed sRNAs recruit the AGO2 protein to the complementary target ncRNAs (i.e., PALRs or TALRs), which alters the transcription status of the *PR* gene via the “gene looping” mechanism (Janowski et al., 2005, 2006, 2007; Schwartz et al., 2008; Younger and Corey, 2009; Yue et al., 2010). This regulatory mechanism is conserved in animals (Janowski et al., 2006; Kim et al., 2006; Han et al., 2007; Schwartz et al., 2008; Napoli et al., 2009; Chu et al., 2010). Two research areas need further investigation: first, whether the plant endogenous PASRs and TASRs function in a similar manner as the manually designed sRNAs reported in animals; and second, whether PALRs and TALRs are recognized as targets of PASRs and TASRs, respectively, in plants. The involvement of TBARs in site-specific DNA methylation has been observed in both plants and animals (Mette et al., 2000; Hawkins et al., 2009; Swiezewski et al., 2009; Zheng et al., 2013; Ma et al., 2017), indicating that it is an important pathway for TBAR-mediated regulation of gene transcription.

## Concluding Remarks and Perspectives

### Need for a Uniform Annotation System for TBAR Research

**FIGURE 1:**
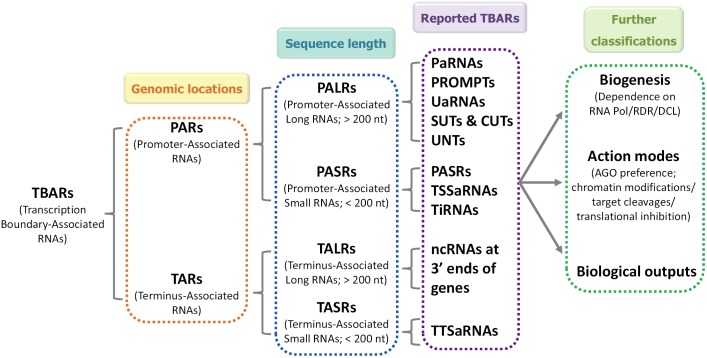
Schematic representation of a uniform annotation system for transcription boundary-associated RNAs (TBARs). In this system, based on their genomic positions, TBARs are first divided into promoter-associated RNA (PARs) and terminus-associated RNA (TARs). Then, according to their sequence length, PARs and TARs are subdivided into long (PALRs and TALRs) and short (PASRs and TASRs) species. For further classification, other important features, such as their biogenesis pathways, modes of action, and biological functions, may be taken into consideration. TBARs, transcription boundary-associated RNAs; PARs, promoter-associated RNAs; TARs, terminus-associated RNAs; PALRs, promoter-associated long RNAs; PASRs, promoter-associated small RNAs; TALRs, terminus-associated long RNAs; TASRs, terminus-associated small RNAs; paRNAs, promoter-associated RNAs; PROMPTs, promoter upstream transcripts; uaRNAs, upstream antisense RNAs; SUTs, stable unannotated transcripts; CUTs, cryptic unstable transcripts; UNTs, upstream non-coding transcripts; TSSaRNAs, transcription start site-associated RNAs; tiRNAs, transcription initiation RNAs; TTSaRNAs, transcription termination site-associated RNAs.

Owing to the heterogeneity of the TBAR population, many aspects of TBARs including sequence characteristics, biogenesis pathways and biological functions are poorly understood. One of the most basic and pressing issues derives from their classification. Some of the TBARs such as PASRs, TSSa-RNAs, and tiRNAs exhibit similar genomic locations and sizes in animals. It is unclear whether these sRNAs belong to distinct classes or to the same class (Jacquier, 2009). It is also unclear whether their biogenesis pathways and action modes overlap. Although some of the TBARs are likely to represent “transcription noise,” increasing cases are being reported that emphasize the non-negligible roles of certain TBARs in gene expression regulation. Hence, a uniform annotation system is required to facilitate in-depth research on TBARs. Some criteria have been proposed previously to determine the classification of PARs, including size ranges and positions relative to the TSSs (Kaikkonen et al., 2011; Lenhard et al., 2012). Here, we propose a preliminary system for TBAR annotation (**Figure 1**). In this system, we first classified TBARs, based on their genomic position, as PARs and TARs. Then, based on their sequence length, both PARs and TARs were divided into long (>200 nt) and short (<200 nt) species. Using this annotation system, most of the recently reported TBARs could be assigned to one of the categories. For further classification, we suggest that additional important features of TBARs, such as biogenesis pathways, modes of action, and biological functions, should be taken into consideration.

### Future Challenges in TBAR Research

To advance our understanding of TBARs, further research is needed in a few areas described below. First, it has been suggested that sRNAs mapped to the transcription boundaries of protein-coding genes are degraded remnants of mRNAs (Schwalb et al., 2016). However, considering the non-random enrichment of TBARs with specific sequence length, such as the animal tiRNAs (18 nt) (Taft et al., 2009a) and plant PASRs and TASRs (23–24 nt) (Ma et al., 2017), at least some of the TBARs are unlikely to be generated through random mRNA decay. Nonetheless, a large proportion of TBARs are subjected to rapid degradation after their maturation. According to previous studies (Davis and Ares, 2006; Chekanova et al., 2007; Preker et al., 2008), mutants of RNA decay pathways (e.g., exosome-depleted cell lines) represent promising options for efficient TBAR cloning.

Second, recent evidence shows that a genetic mutation within a protein-coding locus may affect not only the gene of interest but also the associated ncRNAs. Notably, some of these affected ncRNAs, in turn, alter the chromatin state through long-range interactions (Tufarelli et al., 2003; Wei et al., 2011). Thus, when investigating the biological consequences of a mutation within a specific genomic locus, it is necessary to consider the effects on the associated ncRNAs.

Third, as mentioned above, the high frequency of poly(A) signals on PROMPTs is related to their rapid degradation, which has been proposed as one of the mechanisms to ensure unidirectional transcription elongation (Ntini et al., 2013). Since most of the mammalian promoters are bidirectional (Core et al., 2008; Seila et al., 2008; Sigova et al., 2013), functional studies on the involvement of PARs in transcription determination are necessary. Till date, three key factors have been proposed as important players in transcription determination (Wei et al., 2011), including the nucleotide composition, chromatin modifications of promoters, and the “gene looping” mechanism. The nucleotide composition within the promoter region affects the directionality of the promoter (Engstrom et al., 2006). In yeast, the TATA element imposes a constraint on the direction of transcription initiation (Park et al., 2014). Additionally, chromatin modifications induced by the promoter-associated transcription of the ncRNAs may serve as codes for the orientation of transcription elongation. Moreover, the “gene looping” model suggests that long-range interactions link the promoter to its favored 3′ end, thus determining the orientation of transcription elongation (Miele and Dekker, 2008; Laine et al., 2009; Tan-Wong et al., 2009). However, detailed mechanisms of unidirectional transcription elongation require further investigation.

Fourth, in addition to the TBARs associated with protein-coding genes, whether TBARs exist on lncRNAs needs to be revisited. Conserved secondary structures are detected at the ends of certain lncRNAs (Ponting et al., 2009; Yu et al., 2017); however, whether these local structures serve as precursors of PASRs or TASRs remains elusive.

Overall, investigations into all of the above-mentioned challenging but intriguing research areas are needed. Results of these investigations are expected to increase our knowledge of TBARs.

## Author Contributions

DY, XM, HW, and YM: wrote the manuscript. ZZ, DY, and XM: prepared the figure and table. All authors read and approved the final manuscript.

## Conflict of Interest Statement

The authors declare that the research was conducted in the absence of any commercial or financial relationships that could be construed as a potential conflict of interest.
